# Development and Validation of DIANA (Diabetes Novel Subgroup Assessment tool): A web-based precision medicine tool to determine type 2 diabetes endotype membership and predict individuals at risk of microvascular disease

**DOI:** 10.1371/journal.pdig.0000702

**Published:** 2025-08-05

**Authors:** Viswanathan Baskar, Mani Arun Vignesh, Sumanth C. Raman, Arun Jijo, Bhavadharini Balaji, Nico Steckhan, Lena Maria Klara Roth, Moneeza K. Siddiqui, Saravanan Jebarani, Ranjit Unnikrishnan, Viswanathan Mohan, Ranjit Mohan Anjana

**Affiliations:** 1 Madras Diabetes Research Foundation (ICMR—Collaborating Centre of Excellence), Chennai, Tamil Nadu, India; 2 Algorithm Health, Chennai, Tamil Nadu, India; 3 Faculty of Medicine, TU Dresden, Dresden, Germany; 4 Wolfson Institute of Population Health, Queen Mary University of London, London, United Kingdom; 5 Dr. Mohan’s Diabetes Specialities Centre (IDF Centre of Excellence in Diabetes Care), Chennai, Tamil Nadu, India; McGill University Faculty of Science, CANADA

## Abstract

**Background:**

Previous research has identified four distinct endotypes of type 2 diabetes in Asian Indians, which include Severe Insulin Deficient Diabetes (SIDD), Combined Insulin Resistant and Deficient Diabetes (CIRDD), Insulin Resistance and Obese Diabetes (IROD), and Mild Age-related Diabetes (MARD). DIANA (Diabetes Novel Subgroup Assessment) is an online precision medicine tool that can predict endotype membership of type 2 diabetes and individual risk for retinopathy and nephropathy.

**Methodology:**

The DIANA tool determines subgroup membership using a machine learning model (support vector machine) on T2D subgroups in the Asian Indian population. We used a support vector machine (SVM) model to classify type 2 diabetes patient endotypes, and the model is trained based on k-fold cross-validation. Its performance was compared with an algorithm determined based on conditional pre-determined cut-offs and weights for each clinical feature [age at diagnosis, BMI, waist, HbA_1c_, Serum Triglycerides, HDL-Cholesterol, (C-peptide fasting, C-peptide stimulated) – optional. This study employed local interpretable model-agnostic explanations (LIME) and SHapley Additive exPlanations (SHAP) to demystify the endotype prediction model. A random forest model was built to assess an individual’s risk for nephropathy and retinopathy based on individual risk algorithms.

**Findings:**

The SVM model has relatively high accuracy, specificity, sensitivity, and precision values compared to conditional pre-determined cut-offs 98% vs 63.6%, 99.8% vs 88%, 98.5% vs 65.1%, and 98.7% vs 63.4%. Clinician face value validation of the prediction by the SVM model reported an accuracy, specificity, sensitivity and precision compared to conditional pre-determined cut-offs 97% vs 85%, 95.3% vs 63%, 95.8% vs 73%, and 98.9% vs 66.9%. Additionally, our study demonstrated the impact of features on ML models through LIME and SHAP analyses. The accuracy of the random forest risk prediction model for nephropathy and retinopathy was 89.6% (p < 0.05) and 78.4% (p < 0.05), respectively.

**Conclusion:**

We conclude that, DIANA is an accurate, clinically explainable AI tool that clinicians can use to make informed decisions on risk assessment and provide precision management to individuals with new-onset type 2 diabetes.

## Author summary

Since 2018, research has highlighted the potential of artificial intelligence (AI) in identifying subtypes within Type 2 Diabetes (T2D) to improve risk stratification. AI models have successfully identified phenotypic clusters of T2D that are associated with different outcomes and responses to treatment. However, most existing models predict subtypes at a single point in time and do not provide insights into how risk progresses over time. Our study introduces the AI-powered Diabetes Novel Subgroup Assessment (DIANA) tool, which can predict T2D endotypes and long-term health outcomes. DIANA tool offers a personalized risk trajectory by utilizing simple demographic and biochemical data to predict microvascular complications. This tool enhances risk prediction beyond current stratification methods by using readily available variables, thereby assisting clinicians in delivering tailored, precision diabetes care. DIANA tool has been validated in diverse cohorts across multiple states in India, demonstrating its effectiveness for personalized risk stratification in both primary and secondary care settings. This tool could enable clinicians to identify high-risk T2D patients early on and implement endotype-specific interventions, thereby transforming the current management of T2D.

## Introduction

Diabetes mellitus affects over 537 million adults worldwide, with estimates showing that it will increase to 643 million by 2030 [1]. Among those affected, type 2 diabetes (T2D) constitutes the majority of cases, but T2D presents with significant heterogeneity in terms of clinical manifestations, disease progression, and complications. The challenge of managing T2D is particularly pronounced in highly populated countries like India, where the diversity of patient phenotypes complicates efforts to standardize care.

India has the second-highest number of diabetes cases in the world, with over 101 million cases reported in the ICMR INDIAB study [2]. The incidence of diabetes is also very high in India [3]. Moreover, type 2 diabetes appears at a much younger age [4]. Earlier research has identified four distinct endotypes of T2D in the Asian Indian population, including severe insulin-deficient diabetes (SIDD), insulin resistance and obese diabetes (IROD), combined insulin resistance and deficient diabetes (CIRDD), and mild age-related diabetes (MARD) [5]. Two common subtypes of these, SIDD and MARD, were also seen in the Caucasian population, resembling characteristics similar to the Asian Indian population [6]. Individuals with some of these endotypes tend to progress rapidly to complications, such as retinopathy and nephropathy, without timely intervention [7]. These endotypes have now been validated in other Indian datasets [8], as well as in South Asians in the UK (Pakistanis and Bangladeshis) [9], Chinese population [10], and are gaining attention globally.

In recent years, Artificial intelligence (AI) and machine learning (ML) models have shown great potential in analysing large datasets to better stratify patients and provide precision treatment [11]. These AI-driven tools have been particularly useful in managing diabetes-related complications, including cardiovascular disease, retinopathy, and nephropathy, and automating the screening and detection of macrovascular and microvascular issues [12]. AI-based models, such as those developed by Alix et al. [13], have demonstrated their capability in predicting T2D risk. However, despite AI’s potential, its broader application remains limited, particularly in diverse populations like Asian Indians, where distinct genetic and phenotypic profiles require tailor-made approaches to diabetes management. Challenges such as data standardization and accessibility to AI technology continue to hinder widespread adoption, particularly in real-world clinical settings. Additionally, some healthcare professionals remain hesitant to integrate AI into daily practice due to concerns about workflow adjustments and the reliability of AI models.

Given these challenges and the need for precision tools to predict T2D endotypes and associated complications, this study has two key objectives. The primary aim was to develop and clinically validate the DIANA (**Dia**betes **N**ovel Subgroup **A**ssessment) for predicting T2D endotypes in the Indian population. The secondary aim was to train and test the model to assess individual risk for complications such as retinopathy and nephropathy using clinical biomarkers. Ultimately, the goal was to assess DIANA’s prediction accuracy and clinical usability to improve diabetes management and patient outcomes.

## Materials and methods

### Study design and participants

Data was obtained from the Diabetes Electronic Medical Records (DEMR) of a chain of tertiary care centres for diabetes management in India, with over 6,30,000 patients treated over 30 years. From the total dataset, 80,118 T2D individuals diabetes duration less than 5 years were selected, with all available baseline parameters, namely age, sex, age at diagnosis of diabetes, body mass index (BMI), waist circumference, glycated haemoglobin (HbA_1c_), serum triglycerides, serum cholesterol (high-density lipoprotein, HDL), total cholesterol, serum creatinine, systolic and diastolic blood pressure and retinopathy examination. As reported in our earlier publication, we used K-means clustering to identify T2D endotypes, which were used as a reference to formulate the conditional pre-determined cut-off-based algorithm [4]. Algorithm Health Private Limited helped to set up the technical capability to execute the pre-determined cut-off algorithm (PDCA), where PostgreSQL was utilised as the designated database system. The Python-built algorithm offered a web interface by employing FastAPI’s functionalities for streamlined web development.

### Definitions

Diabetes was diagnosed if the fasting plasma glucose level was ≥ 126 mg/dL (7.0 mmol/L) and/or 2-h post-load glucose level was ≥ 200 mg/dL (11.1 mmol/L) and/or if the patient had been prescribed pharmacotherapy for diabetes by a physician [14]. In contrast, the absence of ketosis diagnosed T2D, good beta-cell reserve as shown by fasting C peptide assay >0.6 pmol/mL, lack of pancreatic calculi (on abdominal radiograph), and response to oral hypoglycemic agents for at least two years [15].

### Retinopathy

A retinal specialist examined the retinal fundus images using four-field stereo colour retinal photography (Model FF 450 plus camera, Carl Zeiss, Jena, Switzerland). by direct and indirect ophthalmoscopy. An Early Treatment Diabetic Retinopathy Study grading system modified and standardised in other population-based studies was used to diagnose retinopathy [16,17].

### Nephropathy

Microalbuminuria was diagnosed if albumin excretion was between 30 and 299 µg/mg, and macroalbuminuria if it was ≥ 300 µg/mg [18]. Nephropathy was defined as either microalbuminuria or macroalbuminuria.

Severe Insulin Deficient Diabetes (SIDD) was characterized by the lowest BMI and waist circumference and the lowest C peptide (fasting and stimulated) levels. HOMA-B and HOMA-IR were both low in this cluster. These individuals had the highest HbA1c values.

Insulin Resistant Obese Diabetes (IROD) was characterized by the highest BMI, waist circumference, and C peptide levels. HOMA-B and HOMA-IR were also the highest for this cluster.

Combined Insulin Resistant and Deficient Diabetes (CIRDD) was characterized by the lowest age at onset and highest triglyceride and HDL cholesterol levels of all four groups. C peptide levels were higher than SIDD but lower than IROD. HOMA-B and HOMA-IR values were also intermediate between SIDD and IROD, indicating the coexistence of insulin deficiency and insulin resistance.

Mild Age-Related Diabetes (MARD) was characterized by a later onset than the other clusters. It was also characterized by the highest HDL cholesterol, fairly preserved C peptide values, and the best metabolic control of all four groups.

### Model development of SVM and pre-determined cut-off algorithm

We used the previously identified four endotypes of T2D in the Asian Indian population [n = 19,084], namely SIDD, IROD, CIRDD, and MARD, as a standard framework [5] for SVM model training, and the same were used for ranking the variable cut-offs for Pre-Determined Cut-off Algorithm (PDCA). We build the SVM model using an RBF kernel to capture non-linear relationships within the data. The hyperparameters, including the penalty parameter (C) and kernel coefficient (gamma), were optimized through grid search with 5-fold cross-validation. Before model training, features were standardized to ensure uniform scaling. A 5-fold cross-validation approach was implemented to evaluate the generalizability of the SVM model. The dataset was randomly partitioned into five equal subsets. Four folds (80% of the data) were used for training, while the remaining one-fold (20%) was used for validation. This process was repeated across five iterations, ensuring that each subset served as the validation set exactly once. Since 5-fold cross-validation inherently rotates validation sets, an independent test set was not used. Instead, model performance was assessed by averaging the accuracy, sensitivity, specificity, and other evaluation metrics across all five validation sets, providing a robust estimate of the model’s predictive capability.

Further, we implemented a Pre-Determined Cut-off Algorithm to classify individuals based on specific biomarker thresholds identified through T2D novel subgroup analysis [4]. Cut-offs were determined using the Consensus-Driven Threshold approach to maximize sensitivity and specificity, integrating clinical guidelines, literature evidence, and statistical distribution analysis to ensure a simple and interpretable decision rule for clinical application. Further, the SVM model was compared with the PDCA, and we evaluated which method was most applicable in clinical practice to determine the T2D endotype accurately.

The cluster centres were chosen based on the significant association with the four T2D endotypes (SIDD, IROD, CIRDD, MARD) and were used to define the weightage of the ranking variable. Univariate logistic regression was applied to assess the association of each clinical parameter (HbA_1c_, BMI, c-peptide (fasting and stimulated), and serum triglycerides), which were used as key ranked variables with high weightage values. Clinical parameters that best characterize each endotype were given a higher ranking. The SIDD was defined by the highest HbA_1c_, lowest c-peptide (fasting and stimulated), and BMI as key variables. Similarly, the MARD group was defined using the lowest HbA_1c_, highest c-peptide (fasting and stimulated), not very high BMI, and serum triglycerides. In contrast, the CIRDD was defined by high HbA1c and highest serum triglycerides, and the IROD was defined based on the highest BMI.

Exclusion criteria were established based on clinically and statistically derived cutoffs to ensure robust endotype differentiation and mitigate classification bias. Clinically irrelevant cut-offs were refined based on real-world clinical presentation, ensuring that thresholds used for variables such as HbA1c, BMI, serum triglycerides, and C-peptide accurately classified endotypes without misrepresenting patients.

Based on the clinical relevancy, for PDCA, the cut-offs were set as follows: SIDD (HbA1c > 8% or BMI < 26 kg/m^2^ in combination with HbA1c > 8) indicating poor glycemic control due to severe β-cell dysfunction and ensuring that patients classified under SIDD predominantly exhibit an insulin-deficient profile rather than insulin resistance.

IROD (BMI > 26 kg/m^2^ and HbA1c > 8%) representing individuals with significant insulin resistance and poor glycemic control, CIRDD (serum triglycerides > 160 mg/dL or HbA1c > 8% in combination with serum triglycerides > 190 mg/dL) reflects both insulin resistance and β-cell dysfunction, MARD (> age of onset 50 years with HbA1c < 8% or those with HbA1c < 8.5%) represents older individuals with relatively preserved β-cell function.

### Model interpretability and explainability

The Support Vector Machine (SVM) model was tested for its interpretability and explainability to increase the clinician’s reliability in making informed decisions from the endotype prediction. Probability estimates of the model were enabled to facilitate interpretability using Local Interpretable Model-agnostic Explanations (LIME). SHapley Additive exPlanations (SHAP) values quantified each feature’s contribution to model predictions, showing positive or negative influences. The iml package wrapped the model, enabling local feature impact analysis. LIME further explained individual predictions by generating data perturbations and fitting a simple surrogate model. This combined approach provided comprehensive insights into both global and local model behaviour.

### Validation of the tool predictability by estimating the accuracy, sensitivity, specificity

1. We validated the predictability of SVM and PDCA on the DEMR dataset (n = 19,084) labelled with T2D endotypes using the k-means clustering as reported in the earlier publication. Validation was performed using the confusion matrix method to accurately estimate the number of predicted events vs actual events. Key estimation metrics used for the validation are accuracy, kappa statistic, sensitivity, specificity, and positive and negative predictive values. The strength of association between the predicted vs validated cohort was determined using Cramer’s V method with a 4 x 4 matrix table. Association estimates were measured using Cramer’s coefficient and Phi coefficient.
2. Ten clinicians with more than 15 years of experience in diabetes practice provided a random set of patient data (n = 450) for face-value validation of the SVM model and pre-determined cut-off algorithm. The clinicians’ recommendations were recorded for these endotypes from both SVM and PDCA as a face-value validation outcome stored as a categorical (yes or no) value.

### Feature selection and risk predictive model

**Fig 1:**
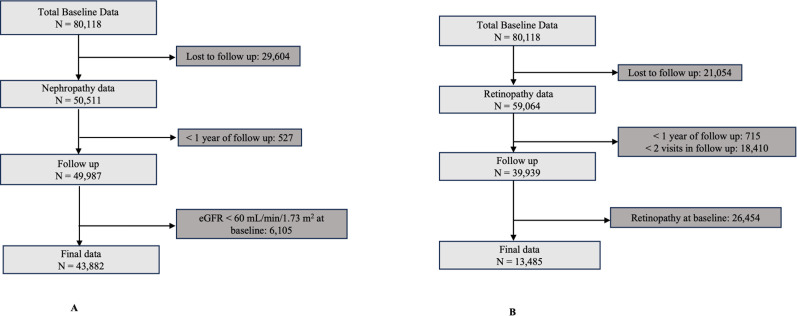
Data selection for microvascular risk prediction model. The figure outlines the data selection process for nephropathy (A) and retinopathy (B) datasets from **80,118** patients from 6,30,000 records from DEMR with complete baseline parameters, including age, sex, age at diabetes diagnosis, BMI, waist circumference, HbA1c, serum lipids, creatinine, blood pressure, and retinopathy examination.

The random forest ensemble method was applied to the random training set and test set of nephropathy (n = 33,032 and n = 10,850) and retinopathy (n = 10,113 and n = 3,372) (Fig 1). Essential features were selected for the model by estimating the mean decrease Gini value. Features were sorted by the highest mean decrease Gini value for the nephropathy cohort (S1 Table) and retinopathy cohort (S2 Table). Features selected with a high mean decrease Gini value are considered to impact the model prediction.

Using the caret package in R, a univariate logistic regression model was used to find the significance of each clinical variable in relation to the risk of retinopathy and nephropathy. The Random forest model was used as the risk prediction model, using the randomForest package in R (version 4.1.3) and the RStudio platform.

### DIANA web interface

DIANA was created with a lightweight web-based RShiny interface and a backend was developed using R programming. It was deployed on an internal server, accessible to intra-network users with clinicians’ and researchers’ login credentials. This web tool handles posts and receives API calls to receive patient data from the Diabetes Electronic Medical Records SQL database, predict the subgroups, and store the prediction outcomes on the SQL server.

## Results

### Validation of DIANA endotypes prediction by SVM model and PDCA

**Fig 2:**
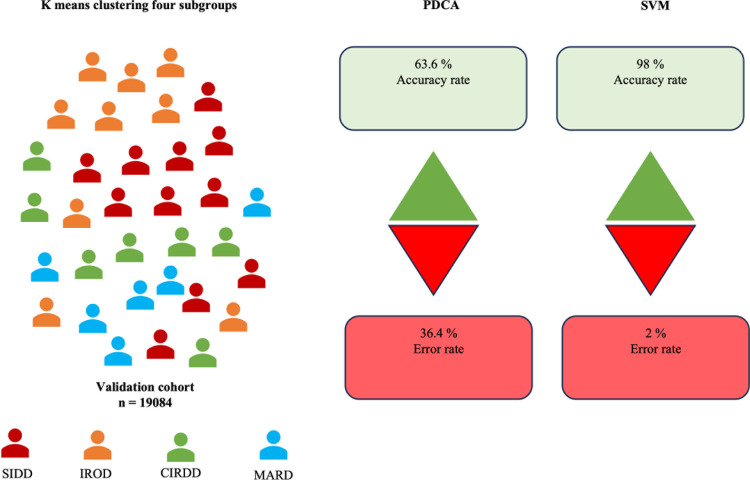
Validation of SVM and PDCA endotype classification using K-means clustering and model performance comparison. The figure illustrates the validation of SVM and PDCA in classifying diabetes endotypes using a k-means clustering approach into four subgroups: SIDD (severe insulin-deficient diabetes), IROD (insulin-resistant obese diabetes), CIRDD (combined insulin-resistant and deficient diabetes), and MARD (mild age-related diabetes), in a validation cohort of 19,084 individuals.

All the patient endotypes predicted by the SVM and pre-determined cut-off algorithm were compared with the original labelled dataset. Validation results show an accuracy rate of SVM vs pre-determined cut-off algorithm (98% vs 63.6%). The SVM model had higher sensitivity, specificity, and precision than PDCA across all the endotypes (Table 1). Predicted results show a high strength of association with the patient-labelled data set, with a Cramer’s V value of 0.79 and 0.54 for SVM and PDCA (Fig 2).

**Table 1:** Performance comparison of SVM and pre-determined cut-off algorithm.

	SVM		PDCA		SVM		PDCA		SVM		PDCA	
Endotypes	95% CI	Specificity	95% CI	Specificity	95% CI	Sensitivity	95% CI	Sensitivity	95% CI	Precision	95% CI	Precision
SIDD	0.99 - 1.00	99.9%	0.84 - 0.86	85.1%	0.98 - 0.99	99.8%	0.85-0.87	86.2%	0.98 - 1.00	100%	0.83 - 0.88	84.4%
IROD	0.97 - 0.99	98.7%	0.84 - 0.85	84%	0.98 - 0.99	99%	0.58 - 0.60	59.2%	0.98 - 0.99	0.98%	0.54 - 0.60	55.8%
CIRDD	0.99 - 1.00	99.6%	0.97 - 0.99	98.8%	0.95 - 0.99	96.4%	0.40 - 0.42	41.1%	0.95 - 0.99	0.98%	0.39 - 0.42	41.1%
MARD	0.97 - 0.99	99.9%	0.81 - 0.84	83.9%	0.98 - 1.00	100%	0.72 - 0.74	74%	0.97 - 1.00	100%	0.71 - 0.74	72.3%

To statistically compare the performance of SVM and PDCA, we employed McNemar’s test to evaluate significant differences in classification accuracy between the two models on paired categorical predictions. Additionally, a paired t-test was conducted on accuracy scores from 5-fold cross-validation to assess differences in the mean performance of the model across validation folds. The p-values from both tests were reported, with p < 0.05 indicating a significant difference in predictive performance.

The SVM model demonstrated high predictive performance across all validation folds. The mean accuracy across the five folds was 99.7% (±0.5%), indicating consistent classification performance. Fold 4 shows the highest accuracy compared to all the folds. Precision, specificity, and sensitivity values across the folds were 98.7% (± 1.1%), 99.8% (± 1.6%), and 98.5% (±1.3%), confirming the model’s ability to effectively distinguish diabetes subtypes better than PDCA whose precision, specificity, and sensitivity (63.4%, 88%, and 65.1%) respectively (Table 1).

### Clinician expert review—Face validation

**Fig 3:**
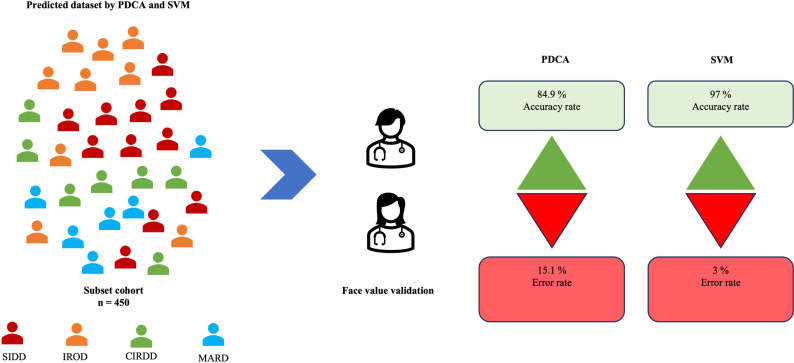
Clinician face value validation of SVM and PDCA for endotype classification. This figure presents the face value validation of the predicted T2D endotypes by SVM and PDCA using a subset cohort of 450 individuals, classified into four groups: SIDD (severe insulin-deficient diabetes), IROD (insulin-resistant obese diabetes), CIRDD (combined insulin-resistant and deficient diabetes), and MARD (mild age-related diabetes).

The clinician face value validation (450 patients, 10 clinicians) for SVM and PDCA showed that 97% and 85% of the clinicians accepted the predicted T2D patient endotypes (Fig 3). Only 3% of clinicians did not agree with the SVM model prediction, which is significantly less than PDCA which is 15%. Face value validation was judged based on each patient’s clinical features and phenotype. Clinician face-value validation demonstrated that 97% of clinicians accepted the SVM model predictions, whereas 85% accepted the PDCA predictions. Clinician face validation results have shown that SVM model had higher precision (98.9%), specificity (95.3%), and sensitivity (95.8%) compared to PDCA had precision (66.9%), specificity (63%), and sensitivity (73%) across all the endotypes (Table 2). A chi-square test was performed on the clinician agreement rates for both models to assess whether this difference was statistically significant. The test yielded a chi-square statistic of 25.4 and a p-value of < 0.001, indicating a significant difference in clinician agreement between the two models. These results confirm that clinicians showed a significantly higher preference for the SVM model over the PDCA model, reinforcing its clinical applicability.

**Table 2:** Performance comparison from Clinician face value of SVM and PDCA for endotype classification.

	SVM		PDCA		SVM		PDCA		SVM		PDCA	
Endotypes	95% CI	Specificity	95% CI	Specificity	95% CI	Sensitivity	95% CI	Sensitivity	95% CI	Precision	95% CI	Precision
SIDD	0.95 - 0.98	97%	0.73 - 0.82	78%	0.92 - 0.96	95%	0.64 - 0.80	78%	0.99 - 1.00	99.4%	0.48 - 0.53	52.3%
IROD	0.96 - 1.00	100%	0.29 - 0.44	36%	0.92 - 0.95	94%	0.46 - 0.66	65%	0.97 - 0.99	98.5%	0.50 - 0.53	52.1%
CIRDD	0.94 - 0.96	94%	0.60 - 0.69	67%	0.93 - 0.96	96%	0.70 - 0.82	72%	0.96 - 0.99	98.1%	0.91 - 0.93	93%
MARD	0.89 - 0.932	90%	0.61 - 0.75	71%	0.97 - 1.00	98%	0.61 - 0.82	77%	0.98 - 0.99	99.7%	0.69 - 0.72	70.2%

### Interpretable machine learning based sub phenotyping of T2D using SHAP and LIME analyses

To improve the interpretability and explainability of the model, we employed SHAP (SHapley Additive exPlanations) and LIME (Local Interpretable Model-agnostic Explanations) to analyze feature contributions in the classification of diabetes endotypes: SIDD, IROD, CIRDD, and MARD. SHAP, which applies random sampling to any dataset with labelled outcomes, was used to understand the importance of global features in predicting diabetes endotypes. The analysis highlighted BMI as the most influential feature, with the highest positive contribution to SIDD (ϕ = 0.33) and the strongest negative impact on IROD (ϕ = -0.36). HbA1c was also a key predictor, positively contributing to SIDD (ϕ = 0.11) but negatively associated with MARD (ϕ = -0.11). C-peptide stimulated (CPS) had a positive influence on SIDD (ϕ = 0.13) but showed negative contributions to CIRDD (ϕ = -0.05) and MARD (ϕ = -0.08). Triglycerides (TGL) positively contributed to IROD (ϕ = 0.03) and negatively to CIRDD (ϕ = -0.04). These results underscore BMI, HbA1c, C-peptide, and lipid parameters as key determinants in differentiating diabetes endotypes, supporting a more refined and personalized risk stratification approach (S1 Fig).

LIME was used to generate a local explanation for an individual prediction, complementing the global interpretability provided by SHAP. To enhance the interpretability of the diabetes subtype classification model, LIME analysis was conducted on two representative patient cases (Case 6286 and Case 51). The results highlight key features influencing the predictions and demonstrate how the model distinguishes between different diabetes subtypes based on clinical parameters.

Case 6286 was predominantly classified as MARD (Mild Age-Related Diabetes) with a 99.80% probability. Key contributing factors included lower HbA1c (≤ 8.3), moderate BMI (≤ 24.3), and older age at onset (> 45.6), suggesting a milder metabolic profile with preserved insulin secretion. In contrast, the absence of insulin deficiency markers led to very low probabilities for SIDD (0.00%), CIRDD (0.20%), and IROD (0.00%), reinforcing the distinction between MARD and more severe insulin-deficient or insulin-resistant subtypes.

Case 51, on the other hand, was classified as SIDD (Severe Insulin Deficient Diabetes) with a high probability of 98.85%. The dominant contributors to this classification included low BMI (≤ 24.3), low C-peptide (CPS ≤ 1.9), high HbA1c (≥ 10.8), and younger age at onset (≤ 37.7), aligning with the characteristics of severe insulin deficiency and poor glycemic control. The model effectively excluded other subtypes, such as MARD (0.03%), CIRDD (0.12%), and IROD (0.00%), as Case 51 lacked obesity-related features and markers of insulin resistance (S2 Fig).

### Univariate logistic regression on microvascular disease biomarkers

Univariate logistic regression was performed to show associations between clinical variables at baseline with risk of nephropathy and retinopathy (Table 3). While all parameters were similar between the cohorts at baseline, results show that male sex, age, diabetes duration, age at onset of diabetes, current age, BMI, blood pressure, waist circumference, glycated haemoglobin, serum triglycerides, HDL & LDL cholesterol, serum creatinine, eGFR and retinopathy status were all significantly associated with the risk of nephropathy. Similarly, male sex, age at onset of diabetes, BMI, diastolic blood pressure, waist circumference, glycated haemoglobin, serum triglycerides, LDL and total cholesterol and serum creatinine at baseline were all significantly associated with the risk of retinopathy.

**Table 3:** Univariate logistic regression of biomarkers in nephropathy and retinopathy cohort.

	Nephropathy				Retinopathy			
Size, n	n = 43882				n = 13483			
Demographic and clinical characteristics	Mean (SD)	Odds Ratios	CI	P value	Mean (SD)	Odds Ratios	CI	P value
Female n (%)	16427 (37.4%)	0.77	0.71 – 0.83	<0.0001 ***	5369 (39.8%)	0.99	0.90 – 1.09	<0.0001***
Male n (%)	27455 (62.6%)				8114 (60.2%)			
Follow up Age, years	55.1 (10.4)	1.10	1.09 - 1.10	<0.0001 ***	55.8 (9.8)	1.07	1.00 - 1.10	<0.0001 ***
Follow up Duration, years	10.9 (7.0)	1.13	1.12 - 1.13	<0.0001 ***	11.3 (5.3)	1.11	1.10 - 1.13	0.004 **
Diabetes duration, years	6.5 (6.2)	1.05	1.04 – 1.05	<0.0001 ***	5.8 (5.3)	1.07	1.06 – 1.08	0.08
Age at onset, years	44.3 (9.2)	1.03	1.02 – 1.03	<0.0001 ***	44.5 (9.0)	0.95	0.95 – 0.96	<0.0001 ***
Age, years	61.3 (10.7)	1.09	1.09 - 1.10	<0.0001 ***	63.3 (10.1)	1.03	1.03 - 1.04	0.9
Waist circumference, cm	95.4 (10.55)	1.00	0.98 – 1.00	0.02 **	94.8 (10.2)	0.98	0.97 - 0.99	<0.0001 ***
BMI, kg/m^2^	27.0 (4.6)	0.99	0.98 – 1.00	<0.0001 ***	27.2 (4.8)	0.96	0.95 – 0.97	<0.0001 ***
Systolic blood pressure, mm Hg	129 (17.0)	1.01	1.01 – 1.02	<0.0001 ***	127 (16.5)	1.00	1.00 – 1.01	0.41
Diastolic blood pressure, mm Hg	81 (9.0)	1.03	1.00 – 1.03	<0.0001 ***	80 (9.0)	1.01	1.00 – 1.02	0.004 **
**Laboratory measurements**								
Glycated hemoglobin, %	9.1 (2.0)	1.10	1.09 – 1.12	<0.0001 ***	8.7 (2.0)	1.15	1.13 – 1.17	<0.0001 ***
Serum triglycerides, mg/dL	176 (94)	1.02	1.00 – 1.03	<0.0001 ***	178 (94.0)	1.00	1.00 – 1.00	<0.0001 ***
HDL Cholesterol, mg/dL	41 (9)	1.00	0.99 – 1.00	<0.0001 ***	40 (9.0)	1.00	0.99 – 1.01	0.07
Serum Cholesterol, mg/dL	183 (41.8)	1.00	0.99 – 1.00	0.25	182 (40.0)	1.00	1.00 – 1.01	<0.0001 ***
LDL Cholesterol, mg/dL	108 (36)	1.00	0.99 – 1.01	<0.0001 ***	107 (35.0)	1.01	1.00 – 1.01	0.002 **
Serum Creatinine, mg/dL	0.78 (0.17)	38.4	32.2 - 45.7	<0.0001***	0.8 (0.22)	1.50	1.40 - 1.80	<0.0001***
Base Line eGFR, mL/min/1.73 m^2^	92.8 (13.77)	0.93	0.92 – 0.93	<0.0001 ***	90.1 (15.5)	0.98	0.98 – 0.99	0.51
Retinopathy Status, n(%)	13032 (29.6%)	2.1	1.93 – 2.41	<0.0001 ***	–			–

### Risk prediction model

The Random Forest (RF) ensemble method was applied to the training sets of nephropathy and retinopathy cohorts, selecting essential features based on the mean decrease in the Gini index (S1 and S2 Tables). The nephropathy model evaluated on the test dataset (n = 11,009) achieved 89.6% accuracy, 98.9% sensitivity, 90.3% PPV, and 64.1% NPV, indicating high positive case detection but limited ability to identify negatives (16.1% specificity). The retinopathy model tested on n = 3,370 yielded 78.4% accuracy, 97.7% sensitivity, 78.8% PPV, and 72% NPV, with similarly low specificity (18.2%) (Table 4). These findings suggest both models are effective for screening purposes but require improvement in identifying negative cases for confirmatory diagnosis.

**Table 4:** Random forest predictive model metrics.

Metrics	Nephropathy model		Retinopathy model	
	95% CI	Accuracy	95% CI	Accuracy
Accuracy	0.88 - 0.89	89.6%	0.75 - 0.79	78.4%
Sensitivity	0.98 - 0.99	98.9%	0.96 - 0.98	97.7%
Specificity	0.12 - 0.17	16.1%	0.18 - 0.21	18.2%
PPV	0.89 - 0.91	90.3%	0.77 - 0.79	78.8%
NPV	0.52 - 0.65	64.1%	0.71 - 0.73	72%

## Discussion

Our study demonstrates the successful application of the DIANA tool in predicting endotypes of type 2 diabetes in an Asian Indian population, with the following key findings: The DIANA tool with the SVM model demonstrated a 98% accuracy compared to the pre-determined cut-off algorithm (PDCA) (63.6%). The overall clinician face value validation of the SVM was 97%, indicating its effectiveness in distinguishing between complex T2D endotypes in real-time clinical practice. Unlike rule-based models like PDCA, SVM does not provide explicit decision rules, which can make direct interpretation challenging for clinicians. However, integrating global and local interpretability techniques enhances transparency and clinical relevance. SHAP analysis highlights BMI, HbA1c, C-peptide, and lipid parameters as key discriminative features for diabetes endotype classification, providing global insights into feature importance. LIME further provides localized explanations of individual predictions, reinforcing subtype distinctions. For example, SIDD patients exhibit lower BMI, low C-peptide, and high HbA1c, while MARD patients are typically older with lower HbA1c levels and a milder metabolic profile. These interpretability methods bridge the gap between predictive accuracy and clinical explainability, increasing clinician trust and the adoption of DIANA in real-world settings.

The findings enhance model explainability, support model transparency, provide clinically meaningful stratification, and advance precision medicine applications in diabetes care. Although inherently interpretable models like rule-based classifiers are considered alternatives, they did not perform well during clinician face-value validation, as their predictive accuracy was significantly lower than that of the SVM model. As a result, there was a trade-off between interpretability and predictive reliability, and DIANA was optimized for high classification accuracy while integrating interpretability techniques to enhance clinical usability.

The DIANA tool demonstrated high sensitivity in predicting the risk of nephropathy and retinopathy, underscoring its potential utility in the early detection of microvascular complications. Using a Random Forest model, DIANA effectively leveraged key clinical biomarkers, including HbA1c, BMI, and serum triglycerides, critical contributors to endotype classification and complication risk prediction.

### AI/ML in T2D endotype prediction

Our identification of four distinct T2D endotypes in the Asian Indian population contributes to the growing literature on machine learning (ML) models for endotype classification. Mizani et al. (2024) achieved a high validation score (F1 > 0.98) in classifying T2D patients into metabolic, early-onset, late-onset, and cardiometabolic subtypes, primarily focusing on long-term outcomes such as hospitalization and mortality [18]. In contrast, our study focuses on the early detection of complications like nephropathy and retinopathy, illustrating the versatility of ML models in addressing both early intervention and long-term prognosis. Our research has demonstrated the use of clinical markers like BMI and HbA_1c_ to classify T2D endotypes with over 94% accuracy, underscoring the adaptability of these models in real-world settings [19].

While the combination of clinical markers and advanced ML models showed great potential, traditional markers (like age, BMI and HbA_1c_) have been shown to outperform complex models in predicting complications [20]. However, our study demonstrates that combining these traditional markers with the ML model balances complexity and practicality, improving precision and enhancing outcomes in diverse T2D populations. This ability to optimize patient stratification using traditional markers and ML techniques aligns with the growing trend of leveraging data-driven methods to refine endotype classification and guide personalized care pathways. Misra et al. (2023) reinforced the utility of data-driven methods, noting that integrating omics data, electronic medical records, and clinical variables improves stratification, helping predict complications and optimize treatment [21]. This aligns with our results, where such models proved effective even with incomplete clinical data.

### Clinical biomarkers in T2D endotype classification

Biomarkers played a critical role in our study’s success, enhancing the precision of endotype classification within the DIANA tool. Using HbA_1c_, BMI, and serum triglycerides significantly contributed to high accuracy for SIDD and CIRDD. This aligns with the findings by Yagin et al. (2023), who reported 91.2% accuracy in classifying diabetic retinopathy (DR) endotypes using metabolomic biomarkers like tryptophan and phosphatidylcholine diacyl [22]. Though Yagin’s study focused on DR, integrating clinical and metabolomic data to improve endotype classification parallels our approach in T2D. Additionally, traditional biomarkers like HbA_1c_ and BMI continue to demonstrate their relevance in predicting T2D outcomes, reinforcing the importance of readily measurable biomarkers in optimizing patient stratification and treatment pathways [23]. Our study further mirrors this by effectively using these markers to predict microvascular complications, such as nephropathy and retinopathy, which validates the robustness of our predictive model. Relying solely on basic clinical features such as age at diagnosis and BMI has limitations in accurately classifying diabetes endotypes, as highlighted in a recent systematic review [21]. However, integrating these traditional clinical markers with advanced machine learning models, as demonstrated by DIANA’s high specificity in population-specific cohorts like CIRDD, enhances the precision of endotype classification and supports the development of personalized diabetes care strategies.

### Utility of predictive models in early detection and management of microvascular complications

Our study confirms that AI models effectively detect microvascular complications, such as nephropathy and retinopathy, before they manifest clinically. Machine learning has been successfully used to detect early ocular microvascular changes, identifying large choriocapillaris flow deficits as early indicators of DR [24]. These findings align closely with our model’s capacity to predict retinopathy risk in T2D patients. Additionally, non-invasive imaging technologies like optical coherence tomography angiography (OCTA) further strengthen predictive models’ precision by providing detailed insights into microvascular health, which is critical to personalized care strategies.

The predictive capabilities of machine learning extend beyond DR to other diabetic complications, including diabetic kidney disease (DKD) and diabetic neuropathy (DN). Models predicting diabetic kidney disease (DKD) have shown promising results, with internal validation indicating a c-statistic of 0.81, supporting their effectiveness in predicting nephropathy risk [25]. However, lower predictive accuracy for DR and DN in some studies underscores the complexity of designing models for these complications, highlighting the need for ongoing refinement. Tools like IDx-DR have shown high sensitivity (87.2%) and specificity (95.5%) in DR detection [26]. In a study using the smartphone-based Remidio ‘Fundus on Phone’ device in India, EyeArt achieved 95.8% sensitivity and 80.2% specificity for detecting DR and 99.1% sensitivity for sight-threatening DR (STDR) [27]. These AI-based tools emphasize proactive management and align with our study’s goal of early detection, especially in resource-limited settings where access to specialized care may be limited.

### Challenges in generalizing AI tools for diabetes care

While AI is promising to enhance diabetes care, several challenges impede its generalizability in real-world settings. One major issue is dataset bias, which may limit the applicability of AI models to underrepresented populations, including variations in ethnicity, age, and gender [28]. In our DIANA tool, the SVM model had 3% clinician face validation, not favouring the correct endotypes. This underscores the need for more inclusive datasets and flexibility in choosing the other endotypes to improve model reliability. Addressing these barriers and ethical concerns, infrastructure limitations, and data privacy issues remains critical for the widespread adoption of AI tools in diabetes management.

In comparison to existing diabetes tools, DIANA introduces several innovations. For instance, the recently published DDZ Diabetes-Cluster-Tool (Acta Diabetologica, 2025) offers a web-based clustering framework primarily designed for European populations [29]. While both DIANA and DDZ implement clustering-based approaches to identify the diabetes endotypes, DIANA differs in several key aspects: it is trained on a large South Asian cohort, integrates both endotype classification and complication risk prediction, and incorporates explainable AI techniques (e.g., SHAP and LIME) to enhance transparency in clinical settings. The population-specific design makes DIANA a pioneering clinical decision-support system tailored for precision diabetes care in India and similar settings.

### Strengths and limitations

The strength of our study is that it underscores the utility of the DIANA tool in identifying distinct endotypes of T2D using easily accessible clinical biomarkers such as HbA_1c_, BMI, serum triglycerides, and cholesterol levels. The algorithm’s high accuracy in predicting endotypes and its ability to forecast nephropathy and retinopathy risks position it as a powerful tool for early detection and personalized care, which are other strengths. However, translating AI models into clinical practice remains a challenge. Early clinical implementation of DIANA across our hospital network has demonstrated its practical utility in guiding treatment decisions. For example, individuals classified into the Severe Insulin-Deficient Diabetes (SIDD) endotype are now being prioritized for early initiation of insulin therapy. Similarly, those identified within the Combined Insulin-Resistant and Deficient Diabetes (CIRDD) group are followed up rigorously with more stringent surveillance for retinopathy and nephropathy. These practices reflect a tangible shift toward phenotype-guided precision care and are beginning to inform individualized treatment strategies. Moreover, structured clinical face validation has shown that clinicians find DIANA intuitive and effective in supporting subtype-based assessment of T2D, compared to previous standard methods. While formal evaluation of DIANA’s impact on long-term treatment outcomes is ongoing, these preliminary observations suggest that the tool is meaningfully influencing clinical workflows and decision-making effectively. While the SVM model performed well in our research and limited clinical settings, real-world validation in independent validation datasets and ongoing refinement by continuous clinical feedback are necessary to ensure consistent accuracy across diverse healthcare environments, which is a limitation. Risk prediction for nephropathy and retinopathy shows less specificity due to data imbalance concerning case and control. This further needs assessment from clinicians, and a feedback system could help fine-tune the risk prediction model. While DIANA predicts an individual’s risk for microvascular complications (nephropathy and retinopathy), it currently does not assess long-term outcomes such as cardiovascular disease (CVD) and mortality. This limitation arises primarily from insufficient longitudinal data to develop and validate robust predictive models for these outcomes. Despite this limitation, DIANA remains a clinically relevant, machine-learning-driven decision-support tool, with future iterations to expand its predictive scope beyond microvascular complications. Future enhancements to DIANA will focus on incorporating longitudinal data to better capture disease progression and enable personalized treatment over time. This will include integrating repeated clinical measurements, medication adjustments, and the evolution of complications to build dynamic risk profiles. Advanced time-aware modeling approaches, such as recurrent neural networks (RNNs), time-series clustering, or survival models, are being explored to predict long-term disease outcomes. In addition, we aim to analyze temporal changes in diabetes endotypes to assess subtype transitions and stability over time, a direction supported by recent evidence showing clinically relevant shifts in subtype trajectories that influence complications and treatment decisions [30]. These upgrades will transform DIANA into a temporally adaptive tool, capable of supporting real-time precision care strategies.

Moreover, the SVM model was trained from a hard clustering labelled dataset, which could be why it performs better over PDCA. This highlights limitations inherent in traditional hard clustering methods, which restrict patients to a single cluster and limit flexibility in accounting for patient overlap between subgroups. This rigidity may have affected the algorithm’s performance during validation. Soft clustering approaches, which offer greater flexibility, remain underexplored in the Asian Indian population and could provide further refinement. Validation of the tool was limited to data derived from the single clustering method, and further development and application of soft clustering could offer additional refinement. While hard clustering provides clear and well-defined patient stratification, it may not fully capture the heterogeneous nature of T2D, where patients often exhibit overlapping metabolic characteristics. Recent studies suggest that soft clustering techniques, such as Gaussian Mixture Models (GMM) and fuzzy c-means clustering (FCM), allow patients to have probabilistic membership across multiple endotypes, improving classification flexibility and disease stratification [31]. A hybrid clustering approach, combining hard clustering for structured classification and soft clustering for probabilistic analysis, has been explored in medical research to enhance diagnostic accuracy, particularly for diseases with continuous progression patterns like diabetes, where strict categorization may lead to misclassification of borderline cases [32,33]. Furthermore, studies have demonstrated that soft clustering improves multimorbidity pattern identification in real-world clinical settings, allowing for more personalized treatment strategies [34]. Given these advantages, future iterations of DIANA will integrate soft clustering methodologies, enabling a more nuanced and flexible classification system that better adapts to patient heterogeneity while maintaining clinical interpretability.

A key limitation of this study is that DIANA has not yet undergone external validation using independent datasets. While we have conducted internal validation through k-fold cross-validation and clinician face-value validation, testing DIANA on new, unseen datasets is essential to assess its generalizability and performance across different patient populations.

Since diabetes endotype distribution and clinical presentations may vary based on demographic, genetic, and environmental factors, external validation would help confirm whether DIANA remains accurate and reliable in diverse clinical settings. To address this, future research will focus on collaborating with external institutions and multi-centre cohorts to validate DIANA on independent datasets. Additionally, we aim to evaluate DIANA’s performance across different ethnic groups to ensure its broad applicability in real-world clinical practice.

Despite this limitation, DIANA presents a clinically relevant, machine-learning-driven approach to personalized diabetes endotype classification and complication risk prediction. Future iterations will enhance its validation and refinement.

## Conclusion

This study underscores the potential of the DIANA tool in accurately identifying T2D endotypes and predicting complications in the Indian population. With its strong performance in distinguishing endotypes like SIDD and CIRDD, the algorithm demonstrated notable predictive power, especially in detecting microvascular complications early. By integrating clinical biomarkers such as HbA_1c_ and serum triglycerides, we enhanced the model’s precision in endotype classification and complication prediction. These findings highlight the transformative role of AI in advancing personalized diabetes management, offering a promising path for early intervention and optimized treatment strategies.

In summary, DIANA serves as a valuable tool for clinicians, enabling informed decisions about treatment and care for individuals with T2D. DIANA uses an SVM model instead of a PDCA to accurately classify an individual’s diabetes endotype and predict the risk of nephropathy and retinopathy. By identifying these risks early, clinicians can implement timely interventions, helping to slow disease progression and improve patient outcomes. Future versions of DIANA will incorporate advanced deep learning techniques and soft clustering techniques, aiming to offer scalable solutions in the diabetic healthcare industry and enhance its utility in diverse populations, potentially saving millions of lives through personalized treatment strategies. Continued feedback and clinician validation of DIANA in broader populations is essential to ensure its clinical utility in real-world settings. DIANA helps clinicians identify T2D endotypes with improved explainability and predicts individual risks of microvascular complications, fostering greater confidence in clinical decision-making.

## Supporting information

S1 Fig Feature contribution analysis using SHAP: Identifying key predictors in endotype classification. (DOCX)

S2 Fig Instance-level explainability using LIME: Understanding individual predictions for endotype classification. (DOCX)

S1 Table Essential features selection for the nephropathy model. (DOCX)

S2 Table Essential features selection for the retinopathy model. (DOCX)
