# Supplementary material for: Development and Validation of DIANA (Diabetes Novel Subgroup Assessment tool): A web-based precision medicine tool to determine type 2 diabetes endotype membership and predict individuals at risk of microvascular disease
Source: PLOS Digit Health. 2025 Aug 5;4(8):e0000702. doi: 10.1371/journal.pdig.0000702 (PMC12324136; doi:10.1371/journal.pdig.0000702)
Supplement: S1 Fig — (DOCX) [file pdig.0000702.s001.docx]

**
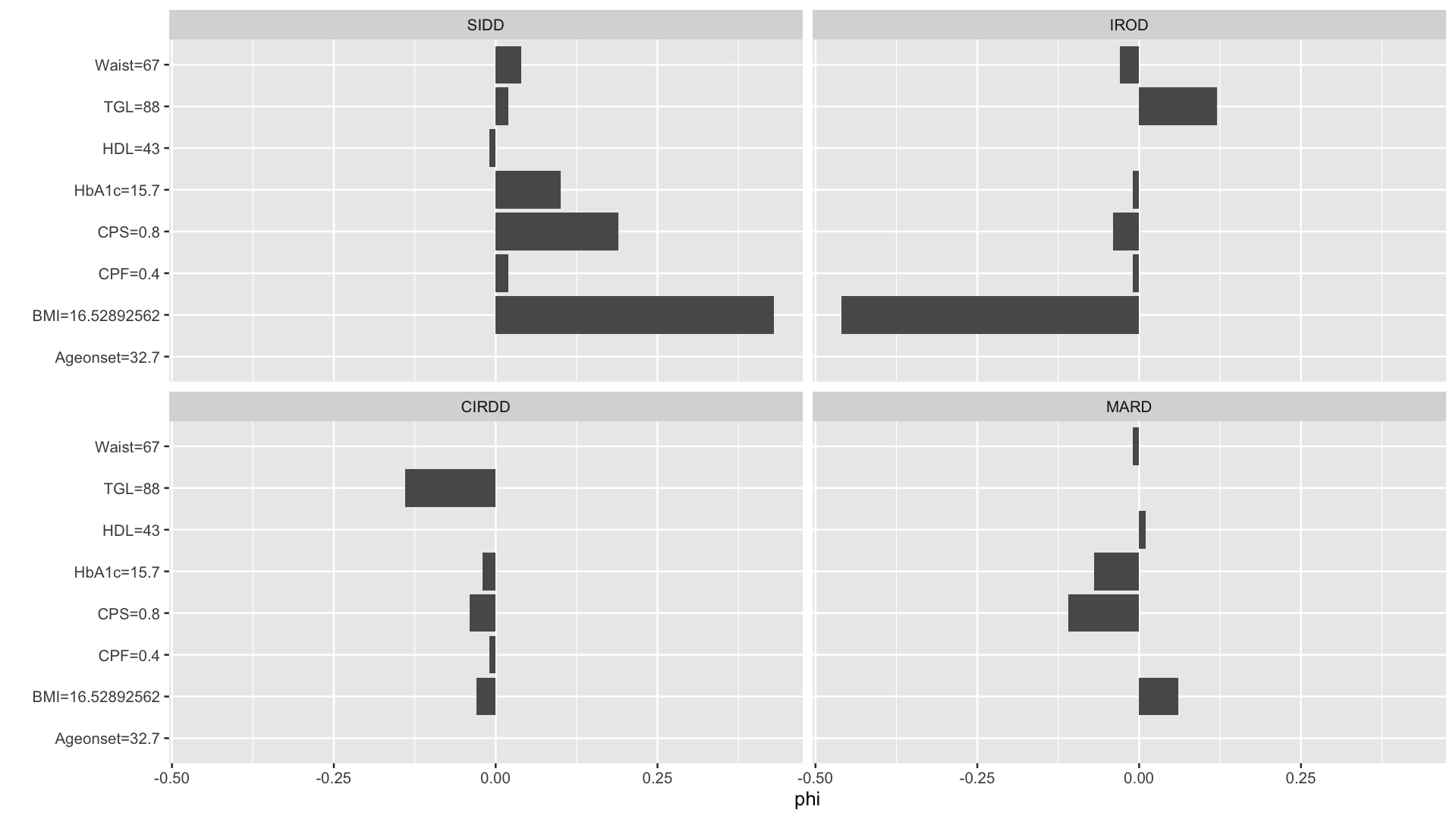
**

S1Fig. Feature Contribution Analysis Using SHAP: Identifying Key Predictors in Endotype Classification
