# Supplementary material for: Development and Validation of DIANA (Diabetes Novel Subgroup Assessment tool): A web-based precision medicine tool to determine type 2 diabetes endotype membership and predict individuals at risk of microvascular disease
Source: PLOS Digit Health. 2025 Aug 5;4(8):e0000702. doi: 10.1371/journal.pdig.0000702 (PMC12324136; doi:10.1371/journal.pdig.0000702)
Supplement: S1 Table — (DOCX) [file pdig.0000702.s003.docx]

S1Table. Essential features selection for the nephropathy model

| **S.No.** | **Features** | **Gini value** |
| --- | --- | --- |
| 1 | Baseline eGFR | 1637.2 |
| 2 | HbA_1c_ | 977.7 |
| 3 | Follow-up duration | 937.3 |
| 4 | Age at onset | 890.4 |
| 5 | Systolic blood pressure | 707.9 |
| 6 | Diabetes duration | 697.5 |
| 7 | Sex | 125.5 |
| 8 | Retinopathy incidence | 115.1 |
