# Supplementary material for: Development and Validation of DIANA (Diabetes Novel Subgroup Assessment tool): A web-based precision medicine tool to determine type 2 diabetes endotype membership and predict individuals at risk of microvascular disease
Source: PLOS Digit Health. 2025 Aug 5;4(8):e0000702. doi: 10.1371/journal.pdig.0000702 (PMC12324136; doi:10.1371/journal.pdig.0000702)
Supplement: S2 Table — (DOCX) [file pdig.0000702.s004.docx]

S2 Table. Essential features selection for the retinopathy model

| **S.No.** | **Features** | **Gini value** |
| --- | --- | --- |
| 1 | Follow-up duration | 759.8 |
| 2 | HbA_1c_ | 425.8 |
| 3 | BMI | 414.1 |
| 4 | Baseline eGFR | 410.3 |
| 5 | Triglycerides | 391.8 |
| 6 | Cholesterol | 370.5 |
| 7 | Age at onset | 340.9 |
| 8 | HDL | 310 |
| 9 | Systolic blood pressure | 255.5 |
| 10 | Sex | 38.1 |
